# Effects of acupoint injection for stroke patients with hemiplegia: A protocol for systematic review and meta-analysis of randomized controlled trials

**DOI:** 10.1097/MD.0000000000028374

**Published:** 2021-12-23

**Authors:** Minghui Liu, Yinyu Wang, Ning Li, Jin Cui, WenRui Fan, Shuo Yang, Li Li, Jie Zeng, Min Li

**Affiliations:** a Guangzhou University of Chinese Medicine, Clinic Medical College of Acupuncture Moxibustion and Rehabilitation, Guangzhou, China; b Medical College of Acupuncture Moxibustion and Rehabilitation, Guizhou University of Traditional Chinese Medicine, China; c Clinical Medical College of Traditional Chinese Medicine, Gansu University of Chinese Medicine, China.

**Keywords:** acupoint injection, hemiplegia, meta-analysis, protocol, systematic review

## Abstract

**Background:**

Acupoint injection has currently received increasing attention as a treatment for hemiplegia. A number of studies have reported that acupoint injection have some advantages in treatment of hemiplegia. However, currently no article has summarized the existing evidence. Our study will evaluate the efficacy and safety of acupoint injection as a clinical treatment for hemiplegia, so that it can provide an important reference for clinical decision-making.

**Methods:**

Randomized controlled trials and case control studies of acupoint injection for hemiplegia according to the included and excluded standard were identified in searches of 6 databases from their inception to February 2021. All data were assessed and extracted by 2 authors independently. The risk of bias assessment recommended by the Cochrane Collaboration was used to assess the quality of the selected studies. Review Manager 5.4 (Cochrane Collaboration) was used to conduct meta-analysis for the efficacy and safety of acupoint injection.

**Result:**

The results of this systemic review and meta-analysis will be submitted to a recognized journal for publication.

**Conclusion:**

This systemic review and meta-analysis will evaluate the efficacy and safety of acupoint injection as a clinical treatment for hemiplegia. We hope this study can make a definitive conclusion for acupoint injection in the treatment of hemiplegia.

**Registration:**

PROSPERO (registration number CRD42021234453).

## 1 Introduction

The global population will contain an estimated 70 million stroke survivors in 2030.^[1]^ A total of 75% of stroke survivors exhibit varying degrees of dysfunction, commonly involving functional impairment of the lower extremities.^[2]^ Neuro-rehabilitation has become a key part of post-stroke treatment, an important component of which is the restoration of lower extremity function.^[3,4]^ However, evidence demonstrating which rehabilitation types best promote mobility in post-stroke patients is scarce.

According to the current practice guidelines, the patients with limb paralysis should get out of bed as soon as possible after their condition is stable, carry out standing and walking rehabilitation training with the aid of instruments, pay attention to muscle strength training of paralyzed limbs, and carry out progressive resistance training for corresponding muscles. Isokinetic muscle strength training can improve the function of paralyzed limbs after stroke, and carry out functional electrical stimulation therapy and electromyographic biofeedback therapy for corresponding muscles at the same time, combined with conventional rehabilitation treatment is also recommended. Acupoint injection is one of the methods of acupuncture.^[5,6]^ In China, it was wildly used to treat stroke patients with hemiplegia to obtain a better curative effect and reduce bad reaction.

Acupoint injection is a supplementary replacement therapy, also known as “water needle,” that involves treating diseases by injecting appropriate medication into relevant acupoints, such as Mingmen (DU 34), Tsusanli (ST 36), and Sanyinjiao (SP6). The theory of traditional Chinese medicine holds that acupoint injection reinforces liver and kidney function and strengthens muscles and bones. At the same time,^[7]^ modern theoretical research also shows that acupoint injection can stimulate the body's meridian system, generate bioelectric activities, and regulate the functions of the viscera and nervous system, increase the energy state, and strengthen the normal metabolic function of the body.^[8]^ Some clinical trials of acupoint injection therapy for hemiplegia have been reported; however, a systematic evaluation of the efficacy of acupoint injection therapy for hemiplegia remains to be conducted. High quality meta-analysis is increasingly regarded as a reliable source of research evidence.^[9–11]^ Therefore, we conducted this systematic review to evaluate the efficacy and safety of acupoint injection as a clinical treatment for stroke patients with hemiplegia.

## 2 Methods

### 2.1 Study registration and ethics

This protocol has been registered at PROSPERO (registration number: CRD42021234453; http://www.crd.york.ac.uk/prospero/display_record.php?ID=CRD42021234453). Data from individual patients will not be used in this systemic review and no privacy will be involved. So the ethical approval is not necessary.

### 2.2 Selection criteria

#### 2.2.1 Type of study

We will include randomized controlled trials (RCTs) and case control studies of acupoint injection for hemiplegia; the language of the literature will not be limited. Controlled clinical trials, case reports, review articles, conference abstracts, editorials, letters, and expert opinions will be excluded.

#### 2.2.2 Participants

Studies enroll patients diagnosed as stroke diagnosis by computed tomography or magnetic resonance imaging and inclusion of patients with unilateral hemiplegia. Characteristics such as age, sex, and ethnicity were not restricted.

#### 2.2.3 Interventions

The experiment group should be applied alone with acupoint injection combined with other basic treatment (electroacupuncture, message, rehabilitation training, etc) the drug types for acupoint injection, the acupuncture points for injection, treatment frequency, and duration of treatment will not be restricted. The control group was treated with basic treatment the same as intervention group.

#### 2.2.4 Outcomes

The primary outcomes is the motor function, the secondary outcomes are score of Fugl-Meyer assessment^[12]^ and the stage of muscle strength, for example, knee joint muscle strength, hip joint muscle strength. The additional outcomes are self care ability of daily life and neurological function.

#### 2.2.5 Search strategy

We will search 6 databases including PubMed, Web of Science, Cochrane Library, Chinese National Knowledge Infrastructure (CHKD-CNKI), Chinese Biomedical Literature Database (CBM), and WanFang Database (Chinese Medicine Premier). We will search all the databases from their inception to February 2021 by 2 independent authors. The search terms “point injection,” “acupoint injection,” and “hemiplegia,” “cerebrovascular disorders,” “stroke,” “paresis.” The medical subject heading terms “hemiplegia,” “acupuncture,” “acupuncture points,” or “injection” will be used.

#### 2.2.6 Data extraction

Data will be extracted by 2 authors independently. All differences will be settled by discussion between the 2 researchers. If we cannot reach an agreement, we will consult a third reviewer. Data extract including the basic information of the trial (name of the first author, year of publication, the country study carried out), basic research information (patient information, experimental intervention, control intervention), evaluation time, outcomes (ADL score,^[13]^ Fugl-Meyer assessment,^[12]^ Ashworth score,^[14]^ etc), and relevant important variables. If information is missing, we contact the authors of the primary studies.

#### 2.2.7 Quality assessment

Assessment of the risk of bias of the included studies will be performed independently by 2 authors and will be ranked as high, low, or uncertain. RCTs will use the Cochrane Collaboration's risk of bias tool, this tool assesses the following domains: random sequence generation, allocation concealment, blinding of participants and personnel, blinding of outcome assessment, incomplete out come data, selective reporting, and other sources of bias. We will rate each domain as low, unclear, or high risk of bias.^[15,16]^ Case control studies will use Newcastle–Ottawa scale. Using that scale, 9 = maximum score, >6 is relatively high quality, 5 to 6 is fair quality, <5 is poor quality.^[17,18]^

#### 2.2.8 Statistical analysis

##### 2.2.8.1 Meta-analysis

We will perform meta-analysis using Review Manager Software 5.4 (Cochrane Collaboration, London, United Kingdom). Dichotomous variables will be calculated as relative risk with 95% confidence interval (CI), and continuous variables will be calculated as the mean difference or standardized mean difference with 95% CI.

##### 2.2.8.2 Measures for heterogeneity

The degree of heterogeneity (*I*^2^) of each outcome will be analyzed using the Chi-squared test, with no significance designate by *P* > .05. *I*^2^ < 50% indicates low heterogeneity of the data and the fixed model adopted for a meta-analysis; otherwise the random effects model will be used. If substantial heterogeneity is detected, subgroup or sensitivity analysis is applied to explore the causes of heterogeneity. If the sources of heterogeneity could not be determined, we adopt descriptive analysis.^[19]^

##### 2.2.8.3 Publication bias

Funnel plot and Egger test will be applied to evaluate the existence of publication bias.

## 3 Discussion

A number of RCTs and case-control studies have shown the efficacy and safety of acupoint injection for the treatment of hemiplegia. While the participants included in the studies were relatively small, in the same time no article has summarized the evidence now existed.

Due to the small simple, we cannot judge the efficacy and safety of acupoint injection accurately. Therefore, we conduct this systemic review and meta-analysis so as to provide reliable evidence for clinical promotion of acupoint injection for stroke patients with hemiplegia. If acupoint injection for hemiplegia is really efficacy and safety, this systemic review could give patients and clinicians several recommendations.

This protocol has been registered, we will strictly execute according to the Cochrane Handbook for Systematic Reviews of Interventions and is presented as per the preferred reporting items for systematic reviews and meta-analyses (PRISMA) guideline.^[20]^ However, there may be several limitations in this study. Firstly, we include only English and Chinese literatures that will be lead to selection bias. Secondly, for most primary studies, the points chose for injection, the treatment frequency and duration of treatment are different, which may cause heterogeneity. Third, the severity of hemiplegia may also affect the results of this study. If the heterogeneity is high, we will conduct a subgroup analysis. Fourthly, our research is based on present research, the emergence of new research in the future may have an impact on current results. So we will update the systematic reviews. In conclusion, we hope this study can make a definitive conclusion for acupoint injection in the treatment of hemiplegia.

## Author contributions

**Conceptualization:** Minghui Liu, Ning Li, Min Li.

**Data curation:** Shuo Yang, Li Li, Wenrui Fan.

**Funding acquisition:** Minghui Liu, WenRui Fan.

**Methodology:** Jin Cui, Shuo Yang, Li Li.

**Project administration:** Yinyu Wang, WenRui Fan.

**Protocol draft:** Minghui Liu, Ning Li.

**Resources:** Minghui Liu, Min Li.

**Study Design:** Minghui Liu, Min Li, and Ning Li.

**Software:** Jie Zeng.

**Validation:** WenRui Fan, Min Li.

**Writing – original draft:** Minghui Liu, Ning Li, Shuo Yang.

**Writing – review & editing:** Min Li.
